# Differential Expression of Human MicroRNAs During Dengue Virus Infection in THP-1 Monocytes

**DOI:** 10.3389/fcimb.2021.714088

**Published:** 2021-09-08

**Authors:** Átila Duque Rossi, Luiza Mendonça Higa, Alice Laschuk Herlinger, Marcelo Ribeiro-Alves, Mariane Talon de Menezes, Ana Lucia Moraes Giannini, Cynthia Chester Cardoso, Andrea T. Da Poian, Amilcar Tanuri, Renato Santana Aguiar

**Affiliations:** ^1^Laboratório de Virologia Molecular, Departamento de Genética, Instituto de Biologia, Universidade Federal do Rio de Janeiro, Rio de Janeiro, Brazil; ^2^Laboratório de Bioquímica de Vírus, Instituto de Bioquímica Médica Leopoldo de Meis, Universidade Federal do Rio de Janeiro, Rio de Janeiro, Brazil; ^3^Laboratório de Pesquisa Clínica em DST/AIDS, Instituto Nacional de Infectologia Evandro Chagas, FIOCRUZ, Rio de Janeiro, Brazil; ^4^Laboratório de Genômica Funcional e Transdução de Sinal, Departamento de Genética, Instituto de Biologia, Universidade Federal do Rio de Janeiro, Rio de Janeiro, Brazil; ^5^Laboratório de Biologia Integrativa, Departamento de Genética, Ecologia e Evolução, Instituto de Ciências Biológicas, Universidade Federal de Minas Gerais, Belo Horizonte, Brazil

**Keywords:** microRNA, monocyte, dengue, gene expression, virus-host

## Abstract

Dengue virus (DENV) is the most widespread arbovirus, responsible for a wide range of clinical manifestations, varying from self-limited illness to severe hemorrhagic fever. Dengue severity is associated with host intense proinflammatory response and monocytes have been considered one of the key cell types involved in the early steps of DENV infection and immunopathogenesis. To better understand cellular mechanisms involved in monocyte infection by DENV, we analyzed the expression levels of 754 human microRNAs in DENV-infected THP-1 cells, a human monocytic cell line. Eleven human microRNAs showed differential expression after DENV infection and gene ontology and enrichment analysis revealed biological processes potentially affected by these molecules. Five downregulated microRNAs were significantly linked to cellular response to stress, four to cell death/apoptosis, two to innate immune responses and one upregulated to vesicle mediated, TGF-β signaling, phosphatidylinositol mediated signaling, lipid metabolism process and blood coagulation.

## Introduction

Dengue is an arthropod-borne viral disease that has rapidly spread around the world in the last decades and for which no antiviral treatment is currently available (Wilder-Smith et al., 2019). It is estimated that half of the world’s population live in areas of risk for dengue and that, annually, from 284 to 528 million new infections occur (Harapan et al., 2020). From these, only about 96 million cases are apparent cases, with symptoms including intense headache, retro-orbital pain, myalgia, arthralgia, high fever, and cutaneous hash, characterizing the mild form of the disease (Harapan et al., 2020). However, infected individuals may develop severe outcomes such as systemic plasma leakage, thrombocytopenia, and severe organ impairment which eventually progress to hypovolemic shock and death (Guzman and Harris, 2016).

Dengue severity is linked to a host intense inflammatory response that leads to increased endothelial permeability and associated hemorrhagic manifestations. Different immune cell types are known to participate in the cytokine storm induced by dengue virus (DENV), including monocytes, macrophages, and dendritic cells (Cerny et al., 2014; Hottz et al., 2014; Alayli and Scholle, 2016; Bhatt et al., 2021). Particularly, an increased number of activated monocytes have been found on blood samples from severe cases suggesting a key role for this cell type during DENV immunopathogenesis (Durbin et al., 2008). Monocytes play a central role in coordinating inflammation both by recruiting macrophages and dendritic cells and by inducing adaptive immunity (Jakubzick et al., 2017). Moreover, monocyte recruitment, activation, and differentiation at the dermis contributes to the establishment of DENV replication sites during early steps of DENV infection (Schmid and Harris, 2014). However, little is known about the mechanisms involved in monocyte responsiveness to DENV at the molecular level.

MicroRNAs are notable for their ability to regulate many different pathways. It is estimated that 60% of human genes expression are regulated by microRNAs. These include genes that play a role on immune response and inflammation (Friedman et al., 2009; Nejad et al., 2018). MicroRNAs have also been described to play a role in cellular strategies to overcome viruses and vice-versa (Trobaugh and Klimstra, 2017). To investigate whether DENV infection regulates microRNAs in monocytes, we assessed the expression pattern of 754-catalogued human microRNAs in the monocytic cell line THP-1 under DENV infection. Eleven cellular microRNAs were considered differentially expressed after infection. Associated pathways were identified by gene ontology analysis and the results suggest the involvement of human microRNAs in different cellular biological processes of known relevance in dengue pathogenesis, as well as others that have not yet been explored in the context of DENV biology. Together, our data suggest a role for human microRNAs during DENV infection in monocytes and highlight interesting candidates for further functional studies.

## Methods

### Virus Propagation

C6/36 mosquito cells (ATCC) were maintained at 28° C in L-15 medium (Gibco) supplemented with 2.95 g/L tryptose phosphate broth (Sigma Aldrich), 0.075% of sodium bicarbonate, 2 mM of glutamine and non-essential amino acids (Gibco), and 5% of inactivated fetal bovine serum (FBS) (ThermoFisher Scietific). C6/36 cells were infected at a multiplicity of infection (MOI) of 0.1 with DENV serotype 2 (DENV-2) (strain 16681). After 9 days of infection, the conditioned medium was harvested, centrifuged at 300 x g for 10 minutes and sterile-filtered through a 0.22 µM membrane (Millipore) to remove cells and cellular debris. Virus stocks were then stored at -80° C. All work involving infectious DENV was performed in a biosafety level (BSL)-3 containment laboratory.

### Virus Titration

Virus titers were determined by plaque assay performed on BHK-21 cells seeded in 12-well plates (maintained at 37°C and 5% CO_2_). Ten-fold serial dilutions of virus stock samples were inoculated into confluent monolayers of BHK-21 cells. After 1 hour, inoculum was removed and semisolid medium (1% carboxymethylcellulose in alpha-MEM supplemented with 1% fetal bovine serum) was added. Cells were further incubated for 5 days prior to formaldehyde-fixation. Cells were stained with crystal violet dye solution for plaque visualization. Titers were expressed as plaque forming units (PFU) per milliliter.

### THP-1 Infection

THP-1 cells (ATCC) were maintained in RPMI 1640 (ThermoFisher Scietific) supplemented with 10% of FBS at 37°C and 5% CO_2_. For infection on six independent experiments, cells were stimulated with phorbol-12-myristate-13-acetate (PMA - Sigma Aldrich) at 15 ηg/mL for 24 hours to induce monocytic activation as previously described (Daigneault et al., 2010). At the time of infection, PMA containing medium was removed and virus samples (DENV-2 16681) were inoculated with an MOI of 10 for 1 hour. Then, non-internalized viruses were removed by PBS washing, and cells were maintained in culture for 24 hours. Finally, cells were harvested to evaluate virus infectivity and microRNA expression.

### Cellular Viability

Viability was evaluated through the capacity of live cells to metabolize resazurin dye (CellTiter Blue, Promega). THP-1 cells were seeded in 96 well plates at a density of 10^4^ cells/well in the presence of PMA 15 ηg/mL for 24 hours. Cells were infected at different MOIs (0.1; 1; 10). Resazurin was added to cells 20- or 44-hours post-infection (for 24h or 48h experiments, respectively). Cellular viability was evaluated by fluorescence emission 4 hours after resazurin addition on a Victor Multilable Plate Reader (PerkinElmer).

### Virus Infectivity

DENV-2 infection was monitored by immunofluorescence assay followed by flow cytometry analysis. Cells intended to flow cytometer analysis were fixed in paraformaldehyde solution (4%) for 15 minutes and permeabilized with triton solution (0.1%) for 20 minutes. Blocking was performed with donkey serum solution (5%) for 1 hour. Cells were incubated for 1 hour with the primary antibody 4G2 obtained from D1-4G2-4-15 hybridoma culture (ATCC). Following primary antibody incubation, cells were PBS washed and stained with the secondary antibody AlexaFluor anti-mouse 488 (Invitrogen) for 1 hour. The number of infected cells was monitored for all six biological replicates with the flow cytometer FACSCalibur (BD Bioscience).

### MicroRNA Extraction, Quality Control and Retro Transcription

Cellular microRNAs were extracted through mirVana™ miRNA Isolation Kit (Thermo Fisher) following manufacturer‘s recommendations. RNA concentration and integrity were evaluated by Agilent RNA 6000 Nano Kit on BioAnalyzer (Agilent Technologies). Only samples with RNA Integrity Number (RIN) superior to nine were considered for further steps. Reverse transcription was performed by TaqMan^®^ microRNA Reverse Transcription Kit (ThermoFisher Scietific) as specified by the manufacturer (with 100ηg of input RNA).

### Large Scale RT-qPCR (OpenArray Platform)

MicroRNA profiles (array plates of 754 microRNAs) from each sample were obtained through RT-qPCR with TaqMan^®^ OpenArray^®^ MicroRNA Panels (ThermoFisher Scietific). Each panel plate holds up to three samples. A total of 6 replicates for each experimental group (mock and DENV-infected cells) were analyzed in study. Sample distribution into panel plates was done with the assistance of the automatic pipetting platform OpenArray^®^ AccuFill™ System (ThermoFisher Scietific). Expression data is publicly available on https://github.com/atilarossi/THP-1_miRs_DENV.

### Real-Time RT-PCR Expression Analysis

From routines created in R language for parsing raw data intensity foreground, background and fractional melting temperature exported from the commercial platform OpenArray^®^ Real-Time PCR System (ThermoFisher Scietific), we made the background correction and the exploratory data analysis (fluorescence accumulation curve graphs of Rn intensity and HRM (High Resolution Melt) for each RT-PCR reaction in real time in different samples. For relative expression, the fluorescence accumulation data of real-time RT-qPCR reactions from each sample were used for fitting four parameter sigmoid curves to represent each amplification curve using the library qPCR for the R statistical package version 3.0.1. The cycle of quantification was determined for each amplification by the maximum of the second derivative of the fitted sigmoid curve. The efficiency of each amplification reaction was calculated as the ratio between the fluorescence of the cycle of quantification and the fluorescence of the cycle immediately preceding that. The estimated efficiency of each microRNA was obtained by the mean of the efficiencies calculated for each amplification reaction of that microRNA. Endogenous controls used in normalization between the different amplified samples were selected by the geNorm method. Normalization factors were calculated as the geometric mean of the expression value of the selected endogenous controls in each sample. The comparisons of means of normalized microRNA expression values between groups were performed by a nonparametric one-way ANOVA with 1,000 unrestricted permutations, followed by post-hoc pairwise comparisons with Bonferroni adjustment by a nonparametric t-test also with 1,000 permutations. Next, we conducted a Type I error adjustment for multiple comparisons by estimating the false positive ratio or false discovery rate (FDR). Results were represented in graphs displaying the microRNA expression levels mean ± standard error. Two-tailed levels of significance ≤ 0.01, 0.05 and 0.1 were considered as “highly significant”, “significant” and “suggestive”, respectively.

### Gene Ontology and Enrichment Analysis

Enrichment analysis of target microRNA genes was performed using the online platform DIANA miRPath v.3 (http://snf-515788.vm.okeanos.grnet.gr). This software considers not only a Gene Ontology database of *in silico* evidence but also incorporates data from experimentally supported targets for tested microRNAs. The enrichment was assessed with a statistical score based on a Fisher’s Exact Test (hypergeometric distribution) adjusted by Benjamini-Hochberg’s False Discovery Rates (FDR) and applying the DAVID’s EASE score for a more conservative statistical analysis. Results that showed p-values ≤ 0.001 were considered significant.

## Results

### DENV Infects PMA-Stimulated THP-1 Cells With High Efficiency and Induces Changes in Cellular MicroRNA Expression Profile

Comparative gene expression studies during infection require a high percentage of infected cells. As activated monocytes are considered susceptible targets of DENV in blood and tissues, THP-1 monocytes were chemically activated through PMA treatment before DENV infection as described in Methods section. To set up the best conditions for high infection rates, activated THP-1 cells were infected at different MOIs, and DENV positive cells were evaluated 24 hours post-infection by immunofluorescence staining and flow cytometer analyses. The highest infection rates (up to 80%) were observed for DENV at the MOI 10 (**Figures S1A, B**). No significant differences in cell viability were observed when mock-infected cells were compared to infected cells at this MOI (**Figure S1C**), ensuring that cell death related pathways would not bias further steps.

**Figure 1:**
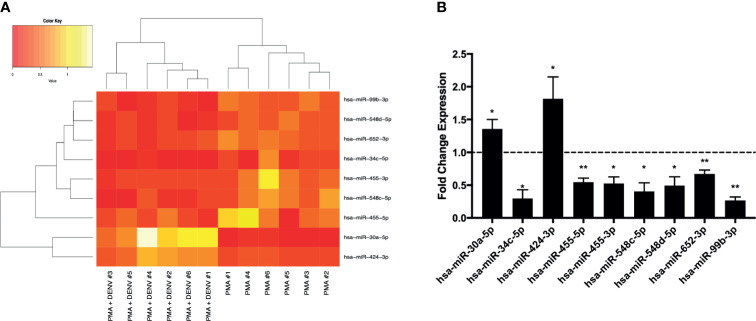
Differential expression of human microRNAs during DENV infection on THP-1 cells (six independent replicates for each experimental group). Expression profile of all infected and mock-infected samples for 9 microRNAs significantly modulated by DENV infection **(A)**. The fold change levels (against mock-infected cells) of regulated microRNAs that showed statistically significant results **(B)**. *p < 0.01 and **p < 0.05.

Six independent experiments of activated THP-1 cells infected with DENV-2 (MOI 10) for 24 hours were then submitted to total RNA extraction followed by RT-qPCR expression analysis of 754 cellular microRNAs. microRNA profiles obtained were compared and, after clusterization, microRNA expression levels were sufficient to discriminate DENV-infected from mock-infected cells (**Figure S2**). DENV significantly induced up-regulation of two microRNAs (hsa-miR-30a-5p and hsa-miR-424-3p) and down-regulated seven microRNAs (hsa-miR-34c-5p, hsa-miR-455-5p, hsa-miR-455-3p, hsa-miR-548c-5p, hsa-miR-548d-5p, hsa-miR-652-3p and hsa-miR-99b-3p) (**Figure 1**). The microRNA hsa-miR-323a-3p was only detectable in infected cells while hsa-miR-489-3p was found exclusively in mock-infected cells (**Figure S3**). Thus, they were classified as upregulated (hsa-miR-323a-3p) and downregulated (hsa-miR-489-3p) by DENV-2.

### Gene Ontology and Enrichment Analysis of DENV-Regulated MicroRNAs Reveal Biological Processes Potentially Altered During Infection

**Figure 2:**
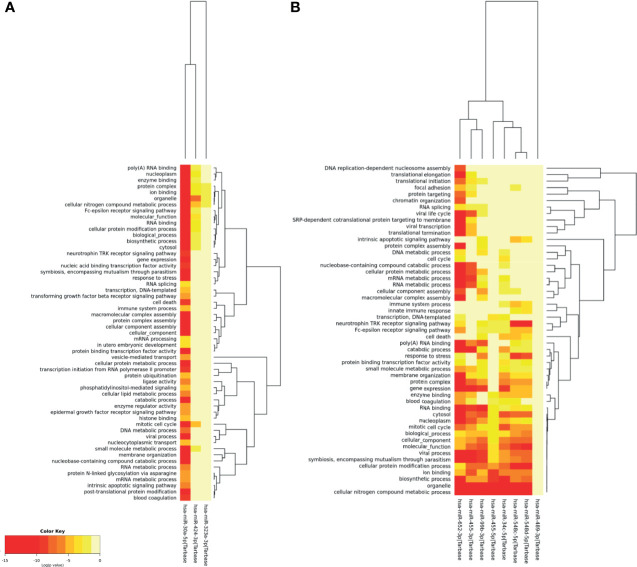
Gene ontology and enrichment analysis for biological processes associated to human microRNAs regulated by DENV infection in monocytes. Gene ontology for up-regulated **(A)** and down-regulated **(B)** microRNAs. Were considered significant results that showed p-values ≤ 0.001.

To identify biological processes targeted by microRNAs modulated during DENV infection, a gene ontology and enrichment analysis (GO) was performed with the software DIANA-miRPath. GO analysis of these 11 microRNAs revealed 55 cellular pathways related to microRNAs up-regulated by DENV (**Figure 2A**) and other 51 pathways linked to microRNAs down-regulated by DENV (**Figure 2B**). Six microRNAs (hsa-miR-30a-5p, hsa-miR-652-3p, hsa-99b-3p, hsa-34c-5p, hsa-miR-548c-5p and hsa-548d-5p) were significantly linked to cellular response to stress, five (hsa-miR-30a-5p, hsa-99b-3p, hsa-34c-5p, hsa-miR-548c-5p and hsa-548d-5p) were linked to cell death/apoptosis, one (has-miR-30a) to vesicle mediated transport and two (hsa-miR-548c-5p and hsa-548d-5p) to innate immune responses. The upregulated hsa-miR-30a showed significant association to biological processes known to be relevant for DENV infection such as TGF-β signaling, phosphatidylinositol mediated signaling, lipid metabolism process and blood coagulation. Downregulated hsa-miR-652-3p, hsa-34c-5p and hsa-miR-548c-5p were also significantly linked to blood coagulation. Biological processes that were never or poorly explored in DENV biology appeared to be linked to DENV-regulated microRNAs, such as SRP-dependent cotranslational targeting membrane system (hsa-miR-652-3p), focal adhesion (hsa-miR-652-3p), Fc-epsilon receptor signaling (hsa-miR-30a-5p, hsa-miR-652-3p, hsa-miR-548c-5p and hsa-548d-5p), and protein N-linked glycosylation (hsa-miR-30a-5p). Interestingly, nine of the eleven regulated microRNAs showed significant GO statistics to cellular nitrogen compound metabolic processes (hsa-miR-30a-5p, hsa-miR-424-3p, hsa-miR-652-3p, hsa-455-3p, hsa-455-5p, hsa-99b-3p, hsa-34c-5p, hsa-miR-548c-5p and hsa-548d-5p), which might indicate the activation of an antiviral state.

## Discussion

The present study explores the expression levels of 754 microRNAs during active DENV infection in an activated monocyte cell model. MicroRNAs are considered a powerful source to unravel molecular pathways behind pathogenesis and promising prognostic biomarkers. Here, we report 11 cellular microRNAs whose expression levels were modulated in response to DENV-2 infection in THP-1 monocytes and *in silico* predicted target pathways for these molecules.

Among microRNAs found to be modulated by DENV herein, some belong to miR families that have already been explored in DENV replication. We describe downregulation of miR-548 family members (hsa-miR548c-5p and hsa-miR548d-5p), which have already been reported to inhibit DENV 1-4 replication (Wen et al., 2015). Authors have shown that endogenous miR-548g-3p suppresses DENV propagation, possibly by direct binding to the Stem Loop A (SLA) promoter in 5’UTR, hindering its interaction with NS5. Likewise, we found hsa-miR-34a-5p to be downregulated in response to DENV-2 infection. Interestingly, miR-34 family members display antiviral activity against flaviviruses, including DENV (Smith et al., 2017). Moreover, miR-34a microRNAs may act as potent activators of interferon (IFN) response due to downregulation of the WNT/β-catenin signaling members WNT2 and WNT3, thereby promoting an antiviral state (Smith et al., 2017). In addition, we found hsa-miR-30a to be up-regulated by DENV-2 infection in monocytes. A same-family microRNA, hsa-miR-30e-3p, is upregulated by DENV-2 infection in HeLa cells suppressing DENV replication by promoting NF-kB-dependent IFN production (Zhu et al., 2014), so it is possible that a similar mechanism takes place in monocytes during DENV infection by hsa-miR-30a, but this needs to be confirmed by further functional studies.

After GO analysis, two upregulated and seven downregulated microRNAs were linked to cellular nitrogen compound metabolic process. This biological process is related to nitrogen compound production and detoxification at the cellular level. Nitrogen compounds are largely involved in many aspects of cellular metabolism during viral infections since nitric oxide plays an important role in innate immunity during viral replication. Nitric oxide and nitric oxide synthase levels have been frequently found elevated during both *in vitro* (monocytes) DENV infections and in clinical samples from infected human subjects (Espina et al., 2003; Neves-Souza et al., 2005; Valero et al., 2013). Our data suggest an involvement of microRNAs in regulation of nitrogen compound metabolism in DENV-infected monocytes supported by a very low p-value on GO analysis.

Another key player in dengue pathogenesis are the Fc receptors, whose interaction with Fc regions of antibodies induces phagocytosis and/or cytokine release. Cytokine production triggered by Fc receptor signaling is mediated by spleen tyrosine kinase (Syk), which is highly active in monocytes during DENV infections *via* antibody-dependent enhancement (ADE) mechanisms (Cheung et al., 2011; Tsai et al., 2014; Callaway et al., 2015). Moreover, Fc receptors are deeply related to these mechanisms since they interact directly with cross-reactive non-neutralizing antibodies bound to viral particles promoting their internalization by a non-canonical mechanism (Boonnak et al., 2011). ADE is part of a well-accepted model to explain DENV immunopathogenesis (Wilder-Smith et al., 2019) and our data suggest that hsa-miR-30a-5p, hsa-miR-548c-5p, hsa-miR-548d-5p and hsa-miR-652-3p could act as Fc-receptor pathway regulators during DENV infection influencing ADE mechanisms.

Several TRKs signaling mechanisms can be involved in modulation of immune responses. For instance, Neurotrophin TRK receptor participates in inflammation and tissue-healing mechanisms by stimulating chemotactic response to CXCL12 by monocyte-derived macrophages (Samah et al., 2008). Activation of this receptor also increases expression of antigen presentation markers (such as HLA-DR and CD80) on human monocyte-derived dendritic cells (Bratke et al., 2007). During viral infection, TRK signaling can also be subverted to promote expression of cellular replication machinery components, enzymes involved in nucleotide synthesis, ribosomal biogenesis, and autophagy regulation, all of which will optimize conditions for viral replication (Hondermarck et al., 2020). In this context, it has been shown that inhibition of fibroblast growth factor receptor 4 (FGFR4) opposingly affects DENV replication and production of infectious virus particles (Cortese et al., 2019).

Hence, these receptors are targeted by viruses to improve their success of an infection, but they can also be pharmacological targets when their exact role is revealed. Although TRK receptors may affect DENV replication and related immune activation, to date, no data is available for their direct involvement in dengue pathogenesis. Our data, bring new insights in this unexplored issue and indicate that hsa-miR-30a-5p, hsa-miR-548c-5p and hsa-miR-548d-5p may act regulating TRK receptor signaling during DENV infection in monocytes.

Blood coagulation is a well-explored biological process in dengue literature since dengue hemorrhagic events are linked to disease severity and mortality rates (Hottz et al., 2018). Although the events that trigger hemorrhagic manifestations are not fully understood, the involvement of a cytokine storm during infection is well described (Guabiraba and Ryffel, 2014). Blood coagulation factors such as prothrombin/thrombin, thrombomodulin, protein C, protein S, fibrinogen/fibrin, and others, are known to be altered during DENV pathogenesis (Azeredo et al., 2015). Our data suggest that hsa-miR-30a-5p, hsa-miR-652-3p, hsa-34c-5p and hsa-miR-548c-5p may be part of this puzzle.

Intriguingly, the upregulated hsa-miR-30a-5p was significantly linked to several biological processes shown by GO analysis. Besides the pathways discussed above, TGF-β receptor signaling pathway appeared to be targeted by this microRNA. TGF-β was previously found at lower levels in dengue patients when compared to healthy donors (Agarwal et al., 1999). Moreover, platelet counts positively correlate with TGF-β levels in DENV infection and genetic polymorphisms in this gene have already been linked to disease severity (Chen et al., 2009; Perez et al., 2010; Malavige and Ogg, 2017). TGF- β is also present on platelet alpha-granules and can be secreted after platelet-monocyte activation and aggregation, frequently found during dengue hemorrhagic events (Singh et al., 2020). Interestingly, hsa-miR-30a-5p has already been found to impact on TGF- β mediated polarization of naïve T cells by reducing transcript levels of IL6R and then affecting Th17 cell differentiation (Schiavinato et al., 2017).

Phosphatidylinositol mediated signaling pathway was also found to be targeted by hsa-miR-30a-5p. Phosphatidylinositol kinases (PI3K) are enzymes with broad cellular signaling functions, including cellular growth, proliferation, differentiation, cell survival and trafficking. Consequently, subversion of PI3K pathways during viral infections are frequent (Hondermarck et al., 2020). Members of this kinase family were already described regulating apoptosis during early steps of flaviviruses’ infection (Lee et al., 2005; Liu et al., 2014), acting on coagulation on dengue pathogenesis (Yeh et al., 2013) and participating on ADE mechanism (Tsai et al., 2014). Here, our data suggest that hsa-miR-30a-5p could also regulate this pathway in monocytes during DENV infection.

Protein N-linked glycosylation was also significantly linked to hsa-miR-30a-5p in this study. N-linked glycosylation participates in virus infectivity and propagation (Mondotte et al., 2007) and the envelope protein harbors two N-glycosylated residues (N67 and N153) that are involved in DENV entry through the interaction with attachment factors on the host cell surface, namely DC-SIGN and mannose receptor (Cruz-Oliveira et al., 2015). NS1 – a non-structural DENV protein mainly involved in immune evasion and critical to DENV replication – has abundant N-glycosylation sites responsible for its secretion and interaction with the complement system (Somnuke et al., 2011; Fan et al., 2014). Although all these findings support the relevance of N-glycosylation in DENV infection, its regulatory mechanisms remain to be elucidated (Zhang et al., 2016). Here, hsa-miR-30a-5p showed up as a candidate regulator of this process.

Finally, downregulated hsa-miR652-3p was the only microRNA for which GO analysis supports a role on SRP-dependent cotranslation protein targeting. The former is a mechanism responsible for protein targeting and direction to the endoplasmic reticulum (ER). As well documented, flaviviruses have their replication sites closely related to the ER. During DENV replication, most of the proteins remain associated with the ER and reside on its lumen until budding *via* Golgi complex (Mukhopadhyay et al., 2005). Although ER targeting seems to be a crucial step for DENV polyprotein translation and viral genome replication, the molecular mechanisms that rely under its synthesis and subcellular organization is not fully understood (Reid et al., 2018). Our data provide evidence of an involvement of SRP-targeting system on DENV replication, where hsa-miR652-3p might play a role.

Taken together, the present data identify eleven human microRNAs whose expression patterns were altered due to DENV infection on a human monocyte cell model. We therefore suggest that these molecules potentially act on DENV replication and/or pathogenesis regulating cellular pathways and host-virus interactions The microRNAs unraveled here are promising candidates for future functional research and their impact on DENV biology and pathogenesis should be further explored.

## Data Availability Statement

The datasets presented in this study can be found in online repositories. The names of the repository/repositories and accession number(s) can be found below: https://github.com/atilarossi/THP-1_miRs_DENV, atilarossi.

## Author Contributions

AR and RA conceived the study. AR, LH, and MM conducted the experiments. AR, LH, MR-A, AG, and CC analyzed the data. AP, AT, and RA financed the study. AR, LH, AH, and AG wrote the manuscript. All authors contributed to the article and approved the submitted version.

## Funding

AP is supported by Fundação Carlos Chagas Filho de Amparo à Pesquisa do Estado do Rio de Janeiro - FAPERJ (201.316/2016; 202.945/2017), Conselho Nacional de Desenvolvimento Científico e Tecnológico - CNPq (309028/2017-5) and La Caixa Bank Foundation (LCF/PR/HR17/52150011). A.T is supported by FAPERJ (E-26/010.001278/2016). RA is supported by CNPq (312688/2017-2; 439119/2018-9) and FAPERJ (202.922/2018).

## Conflict of Interest

The authors declare that the research was conducted in the absence of any commercial or financial relationships that could be construed as a potential conflict of interest.

## Publisher’s Note

All claims expressed in this article are solely those of the authors and do not necessarily represent those of their affiliated organizations, or those of the publisher, the editors and the reviewers. Any product that may be evaluated in this article, or claim that may be made by its manufacturer, is not guaranteed or endorsed by the publisher.

## References

[B1] Agarwal R. Elbishbishi E. A. Chaturvedi U. C. Nagar R. Mustafa A. S. (1999). Profile of Transforming Growth Factor-Beta 1 in Patients With Dengue Haemorrhagic Fever. Int. J. Exp. Path. 80, 143–149. 10.1046/j.1365-2613.1999.00107.x 10469270 PMC2517771

[B2] Alayli F. Scholle F. (2016). Dengue Virus NS1 Enhances Viral Replication and Pro-Inflammatory Cytokine Production in Human Dendritic Cells. Virology 496, 227–236. 10.1016/j.virol.2016.06.008 27348054 PMC4969143

[B3] Azeredo E. L. De, Monteiro R. Q. Pinto L. M. (2015). Thrombocytopenia in Dengue: Interrelationship Between Virus and the Imbalance Between Coagulation and Fibrinolysis and Inflammatory Mediators. Mediators Inflamm. 2015, 313842. 10.1155/2015/313842 25999666 PMC4427128

[B4] Bhatt P. Sabeena S. P. Varma M. Arunkumar G. (2021). Current Understanding of the Pathogenesis of Dengue Virus Infection. Curr. Microbiol. 78 (1), 17–32. 10.1007/s00284-020-02284-w 33231723 PMC7815537

[B5] Boonnak K. Dambach K. M. Donofrio G. C. Tassaneetrithep B. Marovich M. A. (2011). Cell Type Specificity and Host Genetic Polymorphisms Influence Antibody-Dependent Enhancement of Dengue Virus Infection. J. Virol. 85, 1671–1683. 10.1128/JVI.00220-10 21123382 PMC3028884

[B6] Bratke K. Maruschke L. Darowski M. Kuepper M. Braun A. Virchow J. C.. (2007). A Role for the Neurotrophin Receptor TrkB on Maturing Dendritic Cells. J. Neuroimmunol. 189, 88–94. 10.1016/j.jneuroim.2007.07.013 17706795

[B7] Callaway J. B. Smith S. A. McKinnon K. P. de Silva A. M. Crowe J. E. Jr. Ting J. P.-Y. (2015). Spleen Tyrosine Kinase ( Syk ) Mediates IL-1b Induction by Primary Human Monocytes During Antibody-Enhanced. J. Biol. Chem. 290, 17306–17320. 10.1074/jbc.M115.664136 26032420 PMC4498069

[B8] Cerny D. Haniffa M. Shin A. Bigliardi P. Tan B. K. Lee B.. (2014). Selective Susceptibility of Human Skin Antigen Presenting Cells to Productive Dengue Virus Infection. PLoS Pathog. 10, e1004548. 10.1371/journal.ppat.1004548 25474532 PMC4256468

[B9] Chen R. Wang L. Cheng J. Chuang H. Chang J. Liu J.. (2009). Combination of CTLA-4 and TGF β 1 Gene Polymorphisms Associated With Dengue Hemorrhagic Fever and Virus Load in a Dengue-2 Outbreak. Clin. Immunol. 131, 404–409. 10.1016/j.clim.2009.01.015 19269255

[B10] Cheung R. Heyworth P. G. Pierce R. H. Cheung R. Shen F. Phillips J. H.. (2011). Activation of MDL-1 ( CLEC5A ) on Immature Myeloid Cells Triggers Lethal Shock in Mice. J. Clin. Invest. 121, 4446–4461. 10.1172/JCI57682 22005300 PMC3204838

[B11] Cortese M. Kumar A. Matula P. Kaderali L. Scaturro P. Erfle H.. (2019). Reciprocal Effects of Fibroblast Growth Factor Receptor Signaling on Dengue Virus Replication and Virion Production. Cell Rep. 27, 2579–2592. 10.1016/j.celrep.2019.04.105 31141684

[B12] Cruz-Oliveira C. Freire J. M. Conceição T. M. Higa L. M. Castanho M. A. R. B. Da Poian A. T. (2015). Receptors and Routes of Dengue Virus Entry Into the Host Cells. FEMS Microbiol. Rev. 39, 2015, 155–170. 10.1093/femsre/fuu004 25725010

[B13] Daigneault M. Preston J. A. Marriott H. M. Whyte M. K. B. Dockrell D. H. (2010). The Identification of Markers of Macrophage Differentiation in PMA-Stimulated THP-1 Cells and Monocyte-Derived Macrophages. PLoS One 5 (1), e8668. 10.1371/journal.pone.0008668 20084270 PMC2800192

[B14] Durbin A. P. Vargas M. J. Wanionek K. Hammond S. N. Gordon A. Rocha C.. (2008). Phenotyping of Peripheral Blood Mononuclear Cells During Acute Dengue Illness Demonstrates Infection and Increased Activation of Monocytes in Severe Cases Compared to Classic Dengue Fever. Virology 376, 429–435. 10.1016/j.virol.2008.03.028 18452966 PMC2546568

[B15] Espina L. M. Valero N. J. Hernández J. M. Mosquera J. A. (2003). Increased Apoptosis and Expression of Tumor Necrosis Factor-A Caused By Infection of Cultured Human Monocytes With Dengue Virus. Am. J. Trop. Med. Hyg. 68, 48–53. 10.4269/ajtmh.2003.68.48 12556148

[B16] Fan J. Liu Y. Yuan Z. (2014). Critical Role of Dengue Virus NS1 Protein in Viral Replication. Virol. Sin. 29, 162–169. 10.1007/s12250-014-3459-1 24903593 PMC8206285

[B17] Friedman R. C. Farh K. K. Burge C. B. Bartel D. P. (2009). Most Mammalian mRNAs are Conserved Targets of miRNAs. Genome Res. 19, 92–105. 10.1101/gr.082701.108 18955434 PMC2612969

[B18] Guabiraba R. Ryffel B. (2014). Dengue Virus Infection: Current Concepts in Immune Mechanisms and Lessons From Murine Models. Immunology 141 (2), 143–156. 10.1111/imm.12188 24182427 PMC3904235

[B19] Guzman M. G. Harris E. (2016). Dengue. Lancet 385 (9966), 453–465. 10.1016/S0140-6736(14)60572-9 25230594

[B20] Harapan H. Michie A. Sasmono R. T. Imrie A. (2020). Dengue: A Minireview. Viruses 12 (8), 829. 10.3390/v12080829 PMC7472303 32751561

[B21] Hondermarck H. Bartlett W. Nurcomb V. (2020). The Role of Growth Factor Receptors in Viral Infections: An Opportunity for Drug Repurposing Against Emerging Viral Diseases Such as COVID-19? FASEB BioAdv. 2, 296–303. 10.1096/fba.2020-00015 32395702 PMC7211041

[B22] Hottz E. D. Medeiros-de-Moraes I. M. Vieira-de-Abreu A. de Assis E. F. Vals-de-Souza R. Castro-Faria-Neto H. C.. (2014). Platelet Activation and Apoptosis Modulate Monocyte Inflammatory Responses in Dengue. J. Immunol. 193, 1864–1872. 10.4049/jimmunol.1400091 25015827 PMC4137323

[B23] Hottz E. D. Bozza F. A. Bozz P. T. (2018). Platelets in Immune Response to Virus and Immunopathology of Viral Infections. Front. Med. 5, 121. 10.3389/fmed.2018.00121 PMC5936789 29761104

[B24] Jakubzick C. V. Randolph G. J. Henson P. M. (2017). Monocyte Differentiation and Antigen-Presenting Functions. Nat. Rev. Immunol. 17, 349–362. 10.1038/nri.2017.28 28436425

[B25] Lee C. Liao C. Lin Y. (2005). Flavivirus Activates Phosphatidylinositol 3-Kinase Signaling To Block Caspase-Dependent Apoptotic Cell Death at the Early Stage of Virus Infection. J. Virol. 79, 8388–8399. 10.1128/JVI.79.13.8388 15956583 PMC1143730

[B26] Liu Y. Liu H. Zou J. Zhang B. Yuan Z. (2014). Dengue Virus Subgenomic RNA Induces Apoptosis Through the Bcl-2-Mediated PI3k / Akt Signaling Pathway. Virology 448, 15–25. 10.1016/j.virol.2013.09.016 24314632

[B27] Malavige G. N. Ogg G. S. (2017). Pathogenesis of Vascular Leak in Dengue Virus Infection. Immunology 151 (3), 261–269. 10.1111/imm.12748 28437586 PMC5461104

[B28] Mondotte J. A. Lozach P. Amara A. Gamarnik A. V. (2007). Essential Role of Dengue Virus Envelope Protein N Glycosylation at Asparagine-67 During Viral Propagation. J. Virol. 81 (13), 7136–7148. 10.1128/JVI.00116-07 17459925 PMC1933273

[B29] Mukhopadhyay S. Kuhn R. J. Rossmann M. G. (2005). A Structural Perspective of the Flavivirus Life Cycle. Nat. Rev. Microbiol. 3, 13–22. 10.1038/nrmicro1067 15608696

[B30] Nejad C. Stunden H. J. Gantier M. P. (2018). A Guide to miRNAs in Inflammation and Innate Immune Responses. FEBS J. 285, 3695–3716. 10.1111/febs.14482 29688631

[B31] Neves-Souza P. C. F. Azeredo E. L. Zagne S. M. O. Valls-de-Souza R. Reis S. R. N. I. Cerqueira D. I. S.. (2005). Inducible Nitric Oxide Synthase (iNOS) Expression in Monocytes During Acute Dengue Fever in Patients and During *In Vitro* Infection. BMC Infect. Dis. 5, 1–12. 10.1186/1471-2334-5-64 16109165 PMC1208887

[B32] Perez A. B. Sierra B. Garcia G. Aguirre E. Babel N. Alvarez M. (2010). Tumor Necrosis Factor-Alpha, Transforming Growth Factor-β1, and Interleukin-10 Gene Polymorphisms: Implication in Protection or Susceptibility to Dengue Hemorrhagic Fever. Hum. Immunol. 71, 1135–1140. 10.1016/j.humimm.2010.08.004 20732366

[B33] Reid D. W. Campos R. K. Child J. R. Zheng T. Chan K. W. K. Bradrick S. S.. (2018). Dengue Virus Selectively Annexes Endoplasmic Reticulum- Associated Translation Machinery as a Strategy for Co-Opting Host Cell Protein Synthesis. J. Virol. 92, e01766–e01717. 10.1128/JVI.01766-17 29321322 PMC5972907

[B34] Samah B. Porcheray F. Gras G. (2008). Neurotrophins Modulate Monocyte Chemotaxis Without Affecting Macrophage Function. Clin. Exp. Immunol. 151, 476–486. 10.1111/j.1365-2249.2007.03578.x 18190610 PMC2276974

[B35] Schiavinato J. L. S. Haddad R. Saldanha-Araujo F. Baiochi J. Araujo A. G. Scheucher P. S.. (2017). TGF-Beta/atRA-Induced Tregs Express a Selected Set of microRNAs Involved in the Repression of Transcripts Related to Th17 Differentiation. Sci. Rep. 7, 3627. 10.1038/s41598-017-03456-8 28620241 PMC5472579

[B36] Schmid M. A. Harris E. (2014). Monocyte Recruitment to the Dermis and Differentiation to Dendritic Cells Increases the Targets for Dengue Virus Replication. PLoS Pathog. 10, e1004541. 10.1371/journal.ppat.1004541 25474197 PMC4256458

[B37] Singh A. Bisht P. Battacharya S. Guchhait P. (2020). Role of Platelet Cytokines in Dengue Virus Infection. Front. Cell. Infect. Microb. 10, 561366. 10.3389/fcimb.2020.561366 PMC7554584 33102253

[B38] Smith J. L. Jeng S. McWeeney S. K. Hirsch A. J. (2017). A MicroRNA Screen Identifies the Wnt Signaling Pathway as a Regulator of the Interferon Response During Flavivirus Infection. J. Virol. 91, e02388–e02316. 10.1128/JVI.02388-16 28148804 PMC5375670

[B39] Somnuke P. Hauhart R. E. Atkinson J. P. Diamond M. S. Avirutnan P. (2011). N -Linked Glycosylation of Dengue Virus NS1 Protein Modulates Secretion, Cell-Surface Expression, Hexamer Stability, and Interactions With Human Complement. Virology 413, 253–264. 10.1016/j.virol.2011.02.022 21429549 PMC3089955

[B40] Trobaugh D. W. Klimstra W. B. (2017). MicroRNA Regulation of RNA Virus Replication and Pathogenesis. Trends. Mol. Med. 23, 80–93. 10.1016/j.molmed.2016.11.003 27989642 PMC5836316

[B41] Tsai T. Chuang Y. Lin Y. Chang C. Wan S. Lin H.. (2014). Antibody-Dependent Enhancement Infection Facilitates Dengue Virus-Regulated Signaling of IL-10 Production in Monocytes. PLoS Negl. Trop. Dis. 8, e3320. 10.1371/journal.pntd.0003320 25412261 PMC4239119

[B42] Valero N. Mosquera J. Añez G. Levy A. Marcucci R. de Mon M. A. (2013). Differential Oxidative Stress Induced by Dengue Virus in Monocytes From Human Neonates, Adult and Elderly Individuals. PLoS One 8, e73221. 10.1371/journal.pone.0073221 24069178 PMC3775775

[B43] Wilder-Smith A. Ooi E. E. Horstick O. Wills B. (2019). Dengue. Lancet 393, 350–363. 10.1016/S0140-6736(18)32560-1 30696575

[B44] Wen W. He Z. Jing Q. Hu Y. Lin C. Zhou R.. (2015). Cellular microRNA-miR-548g-3p Modulates the Replication of Dengue Virus. J. Infect. 70, 631–640. 10.1016/j.jinf.2014.12.001 25499200

[B45] Yeh T. Liu S. Lin K. Kuo C. Kuo S. Huang T. (2013). Dengue Virus Enhances Thrombomodulin and ICAM-1 Expression Through the Macrophage Migration Inhibitory Factor Induction of the MAPK and PI3K Signaling Pathways. PLoS One 8, e55018. 10.1371/journal.pone.0055018 23383040 PMC3557271

[B46] Zhang R. Miner J. J. Gorman M. J. Rausch K. Ramage H. James P.. (2016). A CRISPR Screen Defines a Signal Peptide Processing Pathway Required by Flaviviruses. Nature 7, 164–168. 10.1038/nature18625 PMC4945490 27383988

[B47] Zhu X. He Z. Hu Y. Wen W. Lin C. Yu J.. (2014). MicroRNA-30e* Suppresses Dengue Virus Replication by Promoting NF-κb-Dependent IFN Production. PLoS Negl. Trop. Dis. 8, e3088. 10.1371/journal.pntd.0003088 25122182 PMC4133224

