# Supplementary material for: Differential Expression of Human MicroRNAs During Dengue Virus Infection in THP-1 Monocytes
Source: Front Cell Infect Microbiol. 2021 Sep 8;11:714088. doi: 10.3389/fcimb.2021.714088 (PMC8455953; doi:10.3389/fcimb.2021.714088)
Supplement: Supplementary file 1 [file DataSheet_1.docx]

Supplementary Material

**Supplementary Figure 1. Activated THP-1 monocytes are highly susceptible to DENV infections. THP-1 cells were chemically activated through PMA treatment and infected with different MOIs of DENV-2 (strain 16681).** Immunostaining for anti-E protein revealed that up to 80% of cells were infected on 24h of infection (A and B). Cell viability assay showed no significant cell death in any conditions tested (C). B and C represent data from six independent experiments.

**Supplementary Figure 2. Expression profile of 754 human microRNAs in DENV-infected or mock-infected THP-1 cells.** MicroRNA expression profile of infected cells is sufficient to discriminate DENV infected from non-infected THP-1 monocytes. Expression foldchange relative to reference gene (RNU48) is represented by heatmap scale as depicted by the color key box.

**Supplementary Figure 3. Raw data for hsa-miR323-3p and hsa-miR489-3p expression.** Four samples amplified for has-miR323-3p on DENV infected THP-1 monocytes while no PMA-treated cells (Mock) showed amplification. Four samples amplified for hsa-miR489-3p on PMA-treated THP-1 monocytes (Mock) while no DENV infected samples showed amplification.
